# Loss of *med14* causes developmental malformations characteristic of VACTERL association by disrupting the Mediator complex

**DOI:** 10.1016/j.gendis.2025.101780

**Published:** 2025-07-24

**Authors:** Jingwen Liu, Xuelai Liu, Feifei Li, Yujie Wang, Liping Yang, Meijia Zhang, Xinyu He, Ting Zhang, Ying Yi Zhang, Qiang Wang

**Affiliations:** a Innovation Centre of Ministry of Education for Development and Diseases, The Sixth Affiliated Hospital, School of Medicine, South China University of Technology, Guangzhou, Guangdong 510006, China; b School of Basic Medicine, Anhui Medical University, Hefei, Anhui 230032, China; c Department of General Surgery, Capital Institute of Pediatrics Affiliated Children Hospital, Beijing 100020, China; d Department of Physiology, Capital Institute of Pediatrics, Beijing 100020, China; e State Key Laboratory of Membrane Biology, Institute of Zoology, University of Chinese Academy of Sciences, Chinese Academy of Sciences, Beijing 100101, China; f Beijing Municipal Key Laboratory of Child Development and Nutriomics, Capital Institute of Pediatrics, Beijing 100020, China

**Keywords:** Disease model, Gene transcription, MED14, Mediator complex, VACTERL association

## Abstract

MED14 is the largest subunit in the Mediator complex and plays a key role in regulating RNA polymerase II-mediated transcription, but its developmental function remains largely unknown. Here, we show that *med14* is required for the proper formation of multi-organs, including the heart, pectoral fin, vertebrae, cloaca, and pronephros, most of which are derived from the mesoderm and are generally affected by VACTERL association, a syndrome with higher prevalence in males. Using this animal model, we further demonstrated that failures in cell fate decisions during organogenesis, but not in early mesoderm formation and subsequent medio-lateral patterning, were the cause of the multi-organ malformations. Importantly, we identified a novel missense mutation in the *MED14* gene (p.Ile550Val) located on the X chromosome in a patient and his family, and neonatal mice carrying the corresponding mutation displayed diagnostic features of VACTERL association. Further investigation revealed that this mutation impairs the structural role of MED14 in the recruitment of specific subunits, such as MED7 and MED17, to the Mediator complex, thereby compromising the interaction between the Mediator complex and RNA polymerase II and decreasing the expression of a set of downstream genes required for the organogenesis of VACTERL-related systems. Taken together, our work uncovers an essential role of MED14 in fetal organogenesis and provides mechanistic insights into the connection between VACTERL association and the Mediator complex.

## Introduction

The Mediator complex is essential for a plethora of transcriptional activities mediated by RNA polymerase II (RNA Poll II) in eukaryotic cells.^1^ Structurally, it comprises four modules: Head, Middle, Tail, and Kinase domain, with the latter present in only some cases.^2^ The Head and Middle modules are crucial for the interaction between Mediator and RNA Pol II, while the Tail module interacts with activators at enhancer regions.^3^ Given its central role in transcription, various subunits of the Mediator complex have been implicated in specific developmental processes. For example, MED12 plays a role in vertebrate neuronal development,^4^ while MED23 is required for placode differentiation during cranial ganglia development.^5^ Genetic variants encoding subunits of the Mediator complex have been linked to a panel of developmental anomalies, such as the deletion of *MED15* in patients with DiGeorge syndrome, as well as missense variants of *MED13L* in patients displaying transposition of the great arteries and intellectual disability.^1^ However, how specific subunits contribute to distinct developmental processes is largely unknown.

As the largest subunit of the core Mediator complex, MED14 serves as a structural and functional backbone for the assembly of the Mediator complex.^7^ Aside from its general role in transcriptional regulation, MED14 is required for a variety of other biological processes, including metabolism and T cell development.^8^^,^^9^ Unique mutant alleles of *med14* have been linked to impaired neural crest development and stem cell maintenance in zebrafish.^10^^,^^11^ However, a thorough characterization of the role (s) of MED14 in development remains to be determined.

Embryonic development is mediated by the intricate orchestration of molecular processes. When any of these events goes awry, severe consequences such as congenital defects may ensue. Clinically, certain congenital defects affecting multiple organs/systems co-occur in a non-random fashion. For example, VACTERL association, historically reported as VATER syndrome in 1973, is defined as the co-occurrence of three or more of the following congenital malformations: vertebral defects, anorectal malformations, cardiac anomalies, tracheo-esophageal fistula with or without esophageal atresia, renal malformations, and limb anomalies.^12^ Its estimated prevalence ranges from 1 in 10,000 to 1 in 40,000 infants.^13^ Affected individuals usually undergo multiple surgical corrections at early stages of their life; and in many cases, their conditions require life-long management.^13^ Familial occurrences suggest that genetic factors contribute to the pathogenesis of VACTERL association.^14^ However, elucidating the underlying genetic causes has been challenging owing to the phenotypic and genotypic heterogeneity of the disorder. Sequencing studies have revealed possible genetic links to VACTERL association, including genetic variants of *FOXF1,*^15^ *HOXD13*,^16^ *PTEN*,^17^ *LPP*,^18^ *HSPA6*,^19^ and *FGF8*^20^; yet direct evidence showing that these genes are causative is lacking.

Despite the complexity of its clinical presentation and underlying etiology, it has not escaped the attention that most of the affected organs arise from the mesoderm.^21^ It is believed that perturbations in mesoderm development drive the pathogenesis of VACTERL association. Yet, due to a lack of animal models, the exact developmental stage and cell lineages being affected are not clear. Another interesting feature of VACTERL association that potentially reveals the underlying etiology is the higher incidence in males,^22^ which implies an X-link inheritance pattern. Variants of an X-linked gene, *Zic3*, were identified in patients diagnosed with VACTERL association.^15^ However, *Zic3* mutant animals failed to reproduce most of the characteristic phenotypes of the disorder.^23^^,^^24^ Hence, the generation of an *in vivo* genetic model that not only recapitulates the pathogenesis but also explains the inheritance patterns of VACTERL association is needed to allow for a comprehensive interrogation of its disease mechanisms.

In this study, we comprehensively described the developmental defects caused by the disruption of Med14 function. By generating and examining animal models of Med14 loss-of-function (LOF) and a novel I550V-MED14 variant identified in the clinic, we revealed that impaired cell fate decisions during organogenesis, rather than failures in early mesoderm formation and subsequent medio-lateral patterning, gave rise to VACTERL-like symptoms. Further investigations showed that the point mutation compromised the interaction of MED14 with MED17 and MED7. In addition, the interaction between RNA Pol II and the Mediator complex was also reduced due to this mutation. Chromatin immunoprecipitation followed by sequencing (ChIP-seq) in conjunction with deep RNA-seq identified downstream genes of MED14 that may be implicated in biological events related to VACTERL association. Overall, our work reveals an essential role of MED14 in organogenesis and elucidates the mechanistic details underlying a potential link between VACTERL association and the Mediator complex.

## Materials and methods

### Ethics statement

The proband, a two-month-old Chinese boy, was admitted to the Department of General Surgery at the Capital Institute of Pediatrics affiliated Children Hospital. He was diagnosed with VACTERL association according to the criteria established by Quan and Smith (1973).^25^ Blood samples were collected from the proband, his parents, and his grandparents. This study received approval from the ethics committees of the Capital Institute of Pediatrics affiliated Children Hospital (Ethics Approval Number: SHERLLM2023054). Written informed consent was obtained from the family for participation in the study and the publication of images in Figure 3, as well as the medical information included in this paper. The animal studies in this study were performed in compliance with the guidelines of the Animal Care and Use Committee of South China University of Technology (Ethics Approval Number: 2023092).

**Figure 1:**
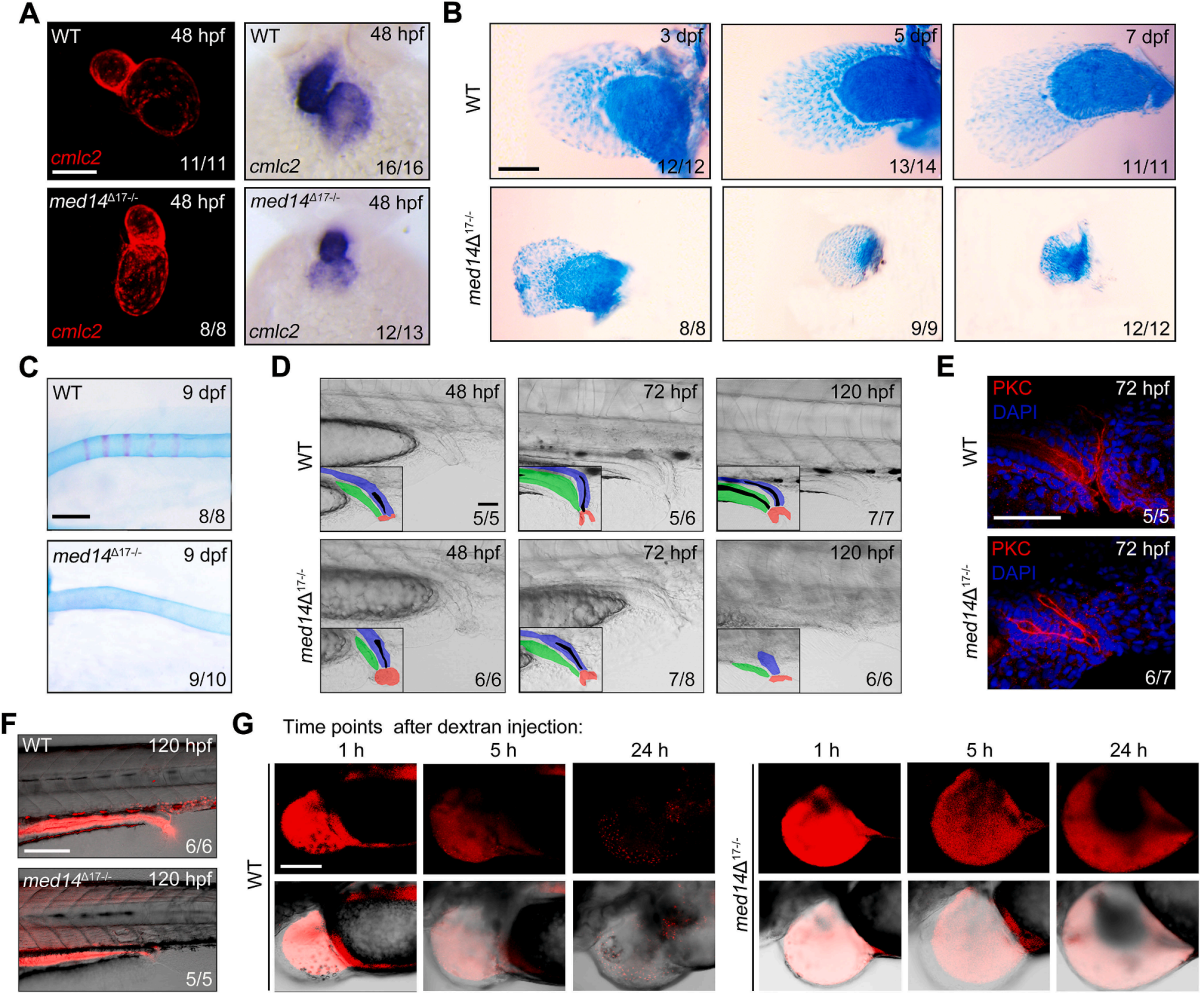
Zebrafish *med14* mutant recapitulates the phenotypes of VACTERL association. **(A)***med14* mutants showed heart looping defects. At 48 hpf, heart tube looping was assessed in *med14* mutants or WT with a Tg(*cmlc2-kalta4-p2a-mCherry*) background (left) or using whole-mount *in situ* hybridization (WISH) with a *cmlc2* probe (right). The ratios of affected embryos were indicated. Scale bar, 50 μm. **(B)** Alcian blue staining in the WT and *med14* mutants from 3 dpf to 5 dpf. Scale bar, 100 μm. **(C)** Alcian Blue and Alizarin Red S staining of WT and *med14* mutants at 9 dpf. The ratio at the bottom-right corner represents the number of embryos with the indicated phenotype versus the total number of embryos examined. **(D)** Live images of cloacae in the WT and *med14* mutant embryos in the lateral view at the indicated time points. The ratio at the bottom-right corner represents the number of embryos with the indicated phenotype versus the total number of embryos examined. **(E)** Immunostaining for α-PKC in WT and *med14* mutants. Nuclei were counter-stained with DAPI. Scale bar, 30 μm. **(F)** Rhodamine dextran excretion assay in *med14* mutants. Rhodamine dextran was injected into the anterior gut of *med14* mutants or WT. The movement and excretion of fluorescent dextran from the gut were monitored 8 h post injection (also see Videos S1, S2). **(G)** Assessment of kidney function in *med14* mutants. WT and *med14* mutants were injected with 40-kDa rhodamine-labeled dextran via the cardiac venous sinus. The fluorescence intensity of each fish was measured at baseline and after 1, 5, and 24 h.

**Figure 2:**
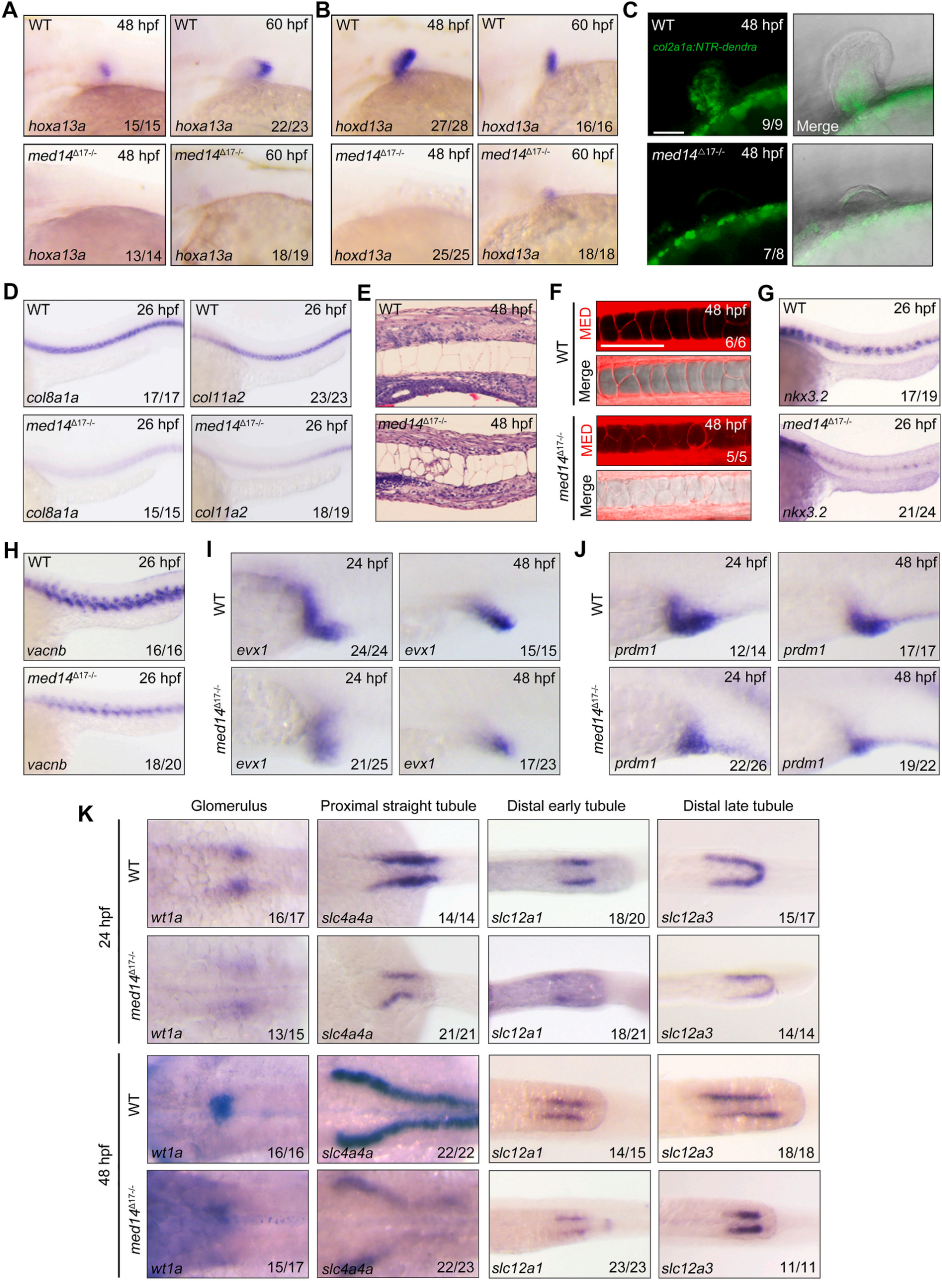
The expression of markers for organogenesis from multiple mesodermal lineages is reduced in *med14* mutants. **(A, B)** Expression of *hoxa13a* (A) and *hoxd13a* (B) in the pectoral fin bud is reduced in *med14* mutants at 48 hpf and 60 hpf. The levels of *hoxa13a* and *hoxd13a* expression were assessed with WISH. **(C)** Representative images of pectoral fins of the WT and mutants in the *Tg(col2a1a:NTR-dendra)* background. The expression pattern of *col2a1a* (left) and an overlay with phase-contrast images (right) of the pectoral fins are shown. Scale bar, 100 μm. **(D)** WISH of *col8a1a* and *col11a2* at 26 hpf in the WT and *med14* mutants. **(E, F)** Longitudinal sections stained with (E) hematoxylin‒eosin (HE) and (F) MED in WT and *med14* embryos at 48 hpf. **(G, H)***nkx3*.2 (G) and *vacnb* (H) expression in WT and *med14* embryos at 26 hpf. **(I, J)** WISH probing for (I) *evx1* and (J) *prdm1* expression in WT embryos and *med14* mutants at 24 hpf and 48 hpf. **(K)** Expression patterns of *wt1a*, *slc4a4a*, *slc12a1*, and *slc12a3* in WT and *med14* mutant embryos at 24 hpf and 48 hpf.

**Figure 3:**
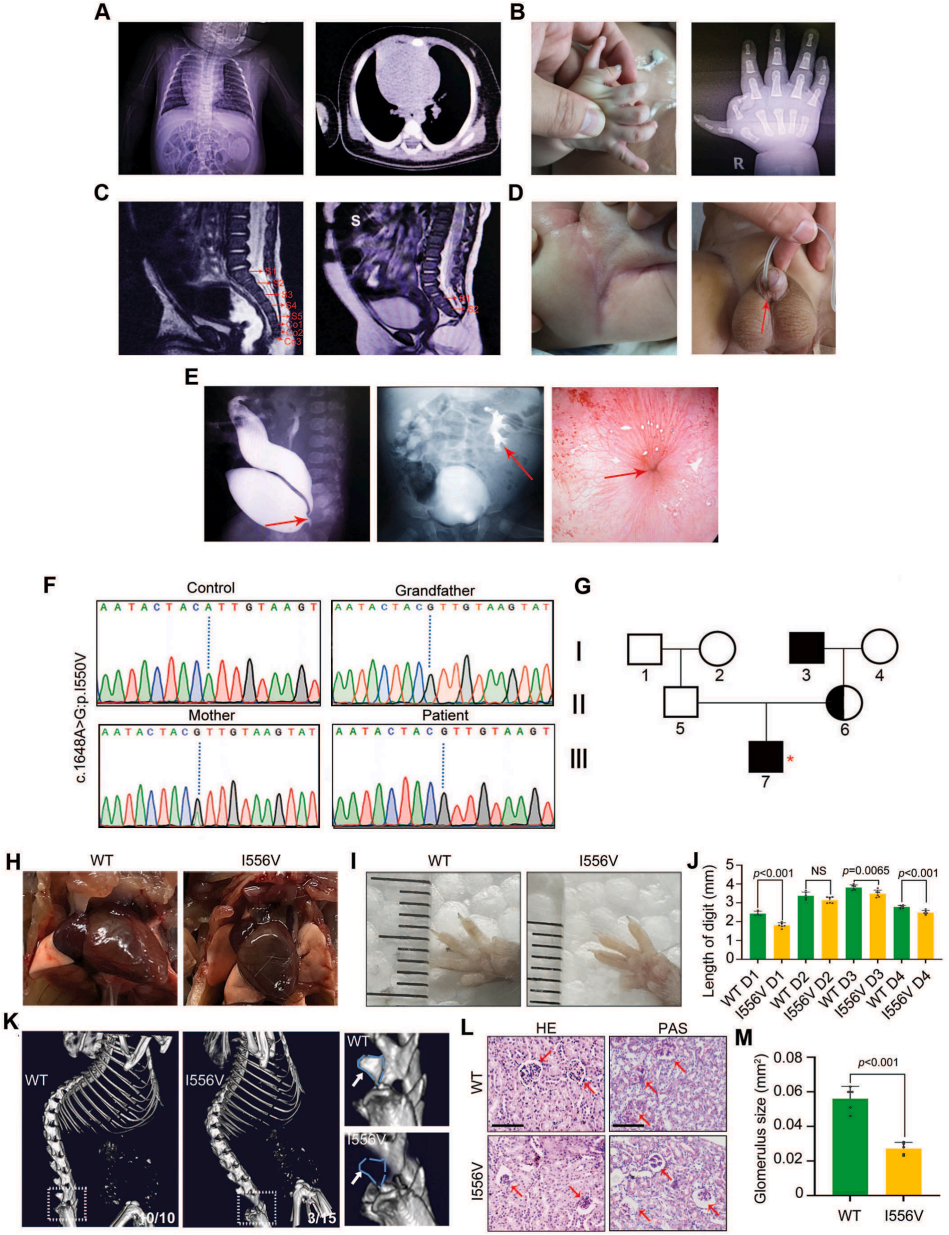
A novel MED14 variant is associated with VACTERL association in a human patient and in a mouse model. **(A)** Mesocardia shown by chest CT. **(B)** Polydactyly of the right hand located on the lateral side of the right thumb, detected by physical examination and X-ray imaging. **(C)** Congenital absence of the sacrococcyx (on the right, with a normal sacrococcyx on the left for comparison) in the sagittal CT image. **(D)** Imperforate anus (left) and hypospadias (right) identified in physical examination. **(E)** Rectourethral fistula, demonstrated by colon contrast examination and simultaneous cystourethrography (left), as well as vesicoureteral reflux shown by cystourethrography (middle), and the orifice of the rectourethral fistula indicated by colonoscopy during the anoplasty (right). **(F, G)** Sanger sequencing validation of the point mutation in the patient, his mother, and his maternal grandfather (F), along with the family pedigree of the patient showing the segregation of the MED14 mutant allele (G), are illustrated. **(H)** Examination of visceral organs in 3-month-old mice revealed mesocardia in I556V mutants. **(I, J)** Digit lengths in WT and I556V MED14 mice were measured 3 months after birth. A representative image of shorten digits in I556V mutants (I) and a summary of measurements (J) are shown, where D1 denotes the digit closest to the bottom of the image, and the *p* values were determined by the unpaired two-tailed Student’s *t*-test. **(K)** Micro-CT experiments showed incomplete formation of the coccygeal vertebrae in about 20% of I556V mutant mice at E18.5. The affected area was zoomed in to provide a better view, with arrows pointing at the missing part of the coccygeal vertebrae (highlighted with a dotted line). **(L)** HE staining (left) and PAS staining (right) at E18.5 indicated a reduction in glomerulus size in some I556V mutant mice. Glomeruli are highlighted with arrows. Scale bar, 100 μm. **(M)** Measurement of glomerulus size in WT and *med14* mutants at E18.5. ∗*P* < 0.05, with *p* values determined by the unpaired two-tailed Student’s *t*-test.

### Exon sequencing and analysis

Genomic DNA was extracted from peripheral blood using a genomic DNA extraction kit (TIANGEN, DP-348-02). Whole-exome sequencing for VACTERL patients was performed by ANGEN Gene Medicine Technology (Beijing, China). Further confirmation of the variant in the entire family was conducted using Sanger sequencing (Majorbio, Shanghai, China).

### Zebrafish strains and maintenance

Wild type (WT), *med14* mutant and *Tg(myl7:kalta4-p2a-mCherry)*, *Tg(col2a1a:NTR-dendra)* zebrafish were reared and maintained according to standard protocols in a recirculating system (ESEN, China). Fish used for the experiments were housed in system tanks. Embryos were collected post natural spawning, incubated at 28.5 °C, and staged based on morphology.

### Zebrafish *med14* mutant generation

In this study, we generated *med14 Δ17* mutants by targeting exon 1 using CRISPR/Cas9. PvuII (NEW ENGLAND Biolabs, R0151L) was employed for *med14 Δ17* genotyping. The target sequence in *med14* gene was 5′-GGAGAGCTTCGTGGGAACAC-3′, with the target region amplified by PCR using the following primers: forward primer: 5′- TTCTATGTACTGTGAAAGATATGACCAG-3′, reverse primer: 5′-ATGACCATTCAAAACAACGAGAAGTC-3′.

### RNA synthesis and microinjection

Capped mRNAs were synthesized using the mMessage mMachine T7 kit (Ambion, AM1344). For the rescue experiments, 80 pg of either WT or mutant med14 mRNA was injected into *med14 Δ17* mutant embryos at the one- or two-cell stage.

### Mouse lines

MED14-I556V mice were generated by GemPharmatech (Jiangsu, China) using the CRISPR/Cas9 gene editing technology. Genotyping of MED14-I556V mice was conducted via Sanger sequencing. All the mice were of the C57BL/6 strain to minimize confounding factors. The mice were housed in microisolator cages with access to a standard chow diet and water, and maintained in an animal colony.

### Cell lines

HEK293T cells were acquired from the American Type Culture Collection (ATCC, USA). Primary MEFs were isolated and cultured following standard protocols.^26^ MEFs were derived from E12.5 embryos of WT and MED14-I556V mice and cultured in Dulbecco’s modified Eagle’s medium (DMEM) supplemented with 10% fetal bovine serum (FBS). All the cells were maintained under standard conditions (37 °C, 5% CO_2_).

### Western blots

The cells were lysed in RIPA buffer (Solarbio Life Science, R0020) containing a protease inhibitor cocktail, and the lysates were kept on ice. The following primary antibodies were used: rabbit anti-MED14 (Thermo Fisher, PA5-79655; 1:1000), rabbit anti-RPB1 (Abcam, ab76123; 1:1000), rabbit anti-MED6 (Thermo Fisher, PA5-65079; 1:1000), rabbit anti-MED7 (Thermo Fisher, PA5-44853; 1:1000), rabbit anti-MED8 (Thermo Fisher, PA5-55829; 1:1000), rabbit anti-MED17 (Thermo Fisher, PA5-84632; 1:800), mouse anti-β-Tubulin (CW, CE0089; 1:5000), and mouse anti-Flag (Abmart, M20008; 1:5000), mouse anti-Myc (MBL, M047-3; 1:5000). Equal amounts of proteins were separated by SDS-PAGE and transferred onto nitrocellulose membranes. The membranes were then incubated with primary antibodies for 3 h at room temperature or overnight at 4 °C. HRP-conjugated secondary antibodies (CW, CW0102 or CW0103; 1:10000) were applied for 1 h at room temperature. Western blot images were scanned and analyzed using ImageJ software for densitometry.

### Co-immunoprecipitation

HEK293T cells, transfected with the designated plasmids via Lipofectamine 2000 (Invitrogen, 11668019), were lysed using RIPA buffer 24 h post-transfection. The cell lysates were subjected to overnight immunoprecipitation at 4 °C with protein A-Sepharose beads (Invitrogen, 101041). Both whole cell lysates and immunoprecipitates were analyzed via western blots.

### Immunofluorescence and confocal microscopy

The embryos were fixed in 4% paraformaldehyde overnight and washed four times with PBST every 5 min. After being blocked in 10% heat-inactivated goat serum for 1 h at room temperature, the embryos were stained with an anti-α-PKC antibody (Santa Cruz, sc-216, 1:200) overnight at 4 °C. After staining, the embryos were washed three times with PBST and incubated with DyLight 594-conjugated goat anti-mouse IgG (Jackson, 715-585-150; 1:200) for 1 h at room temperature. Nuclei were stained with DAPI (Sigma, D9542; 1:10,000) for 10 min. The stained embryos were embedded in 2% low melting agarose and imaged using a Nikon A1R + confocal microscope under identical settings.

### Whole-mount *in situ* hybridization

Antisense RNA probes were synthesized by transcribing linearized plasmids with SP6 or T7 polymerase using a digoxigenin labeling mix (Roche), as described previously.^27^ An alkaline phosphatase-coupled anti-digoxigenin antibody (Roche, 11093274910) was used to localize the hybridized probes. Chromogenic detection was performed using NBT/BCIP (Roche, 11442074001), resulting in blue precipitates for visualization.

### Paraffin section and eosin/hematoxylin staining

For histological analysis, embryos were fixed in 4% PFA overnight. Next, they were then dehydrated in an ethanol series, cleared in xylene, and embedded in paraffin wax. Four-micrometer-thick sections were cut using a microtome (Leica, Germany), stained with hematoxylin and eosin, and imaged using an Axioplan2 microscope (Zeiss, Germany).

### PAS staining

Mouse kidneys were fixed in 4% paraformaldehyde for 24 h, dehydrated, embedded in paraffin, and sectioned for staining with the Periodic Acid Schiff Kit (Solarbio, G1281).

### Alcian Blue and Alizarin Red S staining

At 9 dpf, the fish were fixed in 4% PFA and stained with Alcian Blue and Alizarin Red S to label the cartilage and calcified tissues, respectively. Alcian Blue staining was also used for imaging the pectoral fins of the zebrafish. Images were captured under a Leica stereomicroscope.

### Excretion and filtration assays

For the excretion assay, fluorescent rhodamine dextran (Thermo Fisher, D1816) was injected into the anterior gut of both WT and mutant embryos at around 112 hpf, when the gut was fully formed in WT embryos. Approximately 8 h post injection (120 hpf), live fluorescent images of the embryos were captured. For kidney function assays, tetramethylrhodamine-labeled 40-kDa dextran (Molecular Probes, D1842) was injected into the cardiac venous sinus of each fish. Dye excretion was visualized using a Nikon A1R + confocal microscope.

### MED staining

Zebrafish embryos were stained with 100 μM MED (Invitrogen, C34556) for 1 h, followed by three washes with egg water.

### RT-qPCR

Total RNA was extracted from zebrafish embryos using an RNA prepPure Tissue Kit (Tiangen Biotech, China), and then converted to cDNA using FastQuant RT Super Mix (Tiangen Biotech, China). RT-qPCR was conducted on a LightCycler 480 Real-Time PCR System (Roche, Switzerland) with SYBR Green I Master Mix reagent (Roche, Switzerland). Each transcript was amplified in triplicate, and the mean values were normalized against those of the β-actin control. The primers used in RT-qPCR are listed in Table S2.

### RNA sequencing

RNA sequencing was performed using whole bodies of zebrafish at 48 hpf. Shanghai Biotechnology Corporation (Shanghai, China) conducted the sequencing of the entire library. After filtering, high-quality reads were aligned to the GRCz10.81 zebrafish genome using HISAT2 with default parameters. Stringtie (version: 1.3.0) was used to quantify gene expression and count reads. Differentially expressed genes were identified using edgeR. Enrichment analysis of gene function was performed in the Metascape platform (http://metascape.prg/gp/index.html).

### ChIP-seq and ChIP-PCR

The ChIP-seq experiments were conducted as previously described.^28^ A MED14 antibody (PA5-79655, Thermo Fisher) was used for both ChIP-seq and ChIP-PCR. ChIP-Seq reads were mapped to the GRCz10.81 zebrafish genome by Bowtie2 (v.2.5.0) with a very sensitive configuration. The uniquely aligned fragments with MAPQ ≥ 30 were extracted using SAMtools (v.1.9). Duplicates were removed by Picard tools MarkDuplicates (https://broadinstitute.github.io/picard/, v.2.27.4). ChIP-seq peaks were called using MACS2 with the parameters “-p 0.005”. RPB1 (Abcam, ab76123; 1:1000) and MED17 (Thermo Fisher, PA5-84632; 1:800) antibodies were used for ChIP-PCR. ChIP results were validated by qPCR using a LightCycler96 thermocycler (Roche) and qPCR SuperMix (TransGen Biotech, China) according to the manufacturer’s instructions. The primers used for ChIP-PCR are listed in Table S3.

### Statistics

Numerical data are presented as the mean ± standard deviation (SD), with error bars indicating the SD. Differences between groups were assessed using unpaired Student’s *t*-test and ANOVA. All the statistical analyses were conducted using GraphPad PRISM 7.0 software. A *P*-value <0.05 was considered statistically significant.

## Results

### Zebrafish *med14* mutant displays developmental defects in multiple organs

To determine the potential role(s) of MED14 in embryonic development, we first sought to establish *med14* mutant lines in zebrafish, which is a popular model for vertebrate development. In WT zebrafish, *med14* transcripts are maternally deposited and ubiquitously expressed at early stages of embryonic development; at later stages, *med14* expression is enriched in the midbrain, hindbrain, retina, and pectoral fin, and is detectable in the notochord (Fig. S1). Using CRISPR/Cas9 genome editing, we constructed a *med14* mutant line, *med14 Δ17*, which has a 17-bp deletion resulting in a premature stop codon (Fig. S2A). Genotyping with PvuII endonuclease digestion distinguished homozygous mutants from heterozygotes and WT (Fig. S2B). Western blotting of lysates from WT and mutant embryos confirmed the complete elimination of Med14 proteins in *med14 Δ17* mutants (Fig. S2C). Consistent with previous findings in a null mutant of *med14*,^10^ the *med14 Δ17* homozygous mutants started to exhibit pericardial edema at 24 hpf (Fig. S2D).

To further assess whether heart development was affected in the *med14 Δ17* mutants, we first examined heart looping at 48 hpf in embryos with a *Tg(cmlc2:KALTA2-p2a-mCherr*y) background, which allows for the specific marking and visualization of the cardiac myocardium; this revealed a failure in heart looping in *med14* mutants (Fig. 1A). Whole-mount *in situ* hybridization (WISH) against *cmlc2* at 48 hpf also indicated mesocardia in *med14 Δ17* mutants (Fig. 1A).

We observed shrinkage of the pectoral fin in the mutant embryos. Therefore, we examined whether cartilage development of the pectoral fin was impaired in *med14 Δ17* mutants. Alcian blue staining revealed that while all cartilage elements were present in WT embryos from 3 to 7 dpf, there was a dramatic reduction in the sizes of disc cartilages and fin rays in *med14* mutants (Fig. 1B), suggesting that Med14 is required for proper development of the pectoral fin in zebrafish.

The above observations align with the heart looping and pectoral fin elongation defects previously reported in a *med14* null mutant.^10^ Since *med14* is expressed in the notochord (Fig. S1), we then further tested whether *med14* is required for vertebral development. Chondrogenesis and mineralization, which are crucial for vertebral formation, were assessed in *med14 Δ17* mutants using Alcian Blue and Alizarin Red S staining, respectively. Evidently, vertebral chondrogenesis was unaffected in the mutants. In contrast, we observed a dramatic reduction in Alizarin Red S staining, indicating disrupted mineralization in the mutant embryos (Fig. 1C).

One of the most well-known congenital conditions that involves developmental defects in multiple organs is VACTERL association, which is defined as the non-random co-occurrence of three or more of the following congenital malformations: vertebral defects, anorectal malformations, cardiac anomalies, tracheo-esophageal fistula with or without esophageal atresia, renal malformations, and limb anomalies.^12^^,^^13^ We observed congenital malformations in the heart, pectoral fin, and vertebrae in the *med14 Δ17* mutants; therefore, we contemplated the possibility that MED14 might be involved in the etiology of VACTERL association. To further assess the development of other relevant organs, we first examined the formation of the cloaca in these mutants. In zebrafish, the posterior gut reaches and fuses with the cloaca during development, leading to the external opening of the gastrointestinal (GI) tract near the pronephric duct.^*29*^ Live imaging showed that the loss of Med14 resulted in blockade of the cloaca (Fig. 1D). Immunofluorescence targeting PKC to stain the GI tract confirmed these blockages in the cloaca and suggested possible narrowing of the hindgut in *med14* mutants (Fig. 1E). To assess cloaca function, we examined the excretion of fluorescent rhodamine dextran at 5 dpf, a stage when the gut begins to excrete waste.^29^ Fluorescent rhodamine dextran injections allowed the visualization of gut peristalsis as the dye moved posteriorly in WT embryos. However, unlike in the WT, where fluorescent dextran was efficiently excreted via the cloaca, *med14* mutants were unable to eliminate dextran via the cloaca (Fig. 1F and Movies S1, S2), indicating that the proper opening of the cloaca requires Med14.

Supplementary video related to this article can be found at https://doi.org/10.1016/j.gendis.2025.101780.

The following are the supplementary data related to this article:

Multimedia component 4 **Movie S1** Rhodamine dextran excretion assay in WT zebrafish embryos. Rhodamine dextran was injected into the anterior gut of WT embryos at 5 dpf, and the movement of fluorescent dextran from the gut was monitored.4 Multimedia component 4

Multimedia component 5 **Movie S2** Rhodamine dextran excretion assay in *med14 Δ17* mutant embryos. Rhodamine dextran was injected into the anterior gut of *med14 Δ17* mutant embryos at 5 dpf and the movement of fluorescent dextran from the gut was monitored. Multimedia component 5

Multimedia component 6 **Movie S3** Cloaca function measured by the excretion of rhodamine dextran in *med14* mutant embryos expressing GFP. Multimedia component 6

Multimedia component 7 **Movie S4** Cloaca function measured by the excretion of rhodamine dextran in med14 mutant embryos expressing WT hMED14. Multimedia component 7

Multimedia component 8 **Movie S5** Cloaca function measured by the excretion of rhodamine dextran in *med14* mutant embryos expressing hMED14-I550V. Multimedia component 8

To assess renal development in our zebrafish model, we conducted a dextran-rhodamine clearance assay to evaluate glomerular filtration, as reported in previous studies.^30^^,^^31^ Upon intracardiac injection of dextran-rhodamine, the pronephric glomerulus in WT embryos was able to clear the dextran moiety from the circulation at 24 h post-injection, while *med14* mutants showed substantial accumulation of fluorescence in the pericardial region (Fig. 1G), suggesting that loss of Med14 disrupts pronephros functions. Taken together, these findings underscore the vital role of Med14 in the formation and function of multiple organs that are generally affected in VACTERL association.

### Loss of *med14* results in impaired cell fate decisions during organogenesis

During vertebrate embryogenesis, the newly formed mesoderm is medio-laterally patterned into axial, paraxial, intermediate, and lateral plate mesoderm, ultimately giving rise to different cell and tissue types that contribute to various organs.^32^ Based on our observations, most of the affected organs in *med14 Δ17* mutants arise from the mesoderm. However, it remains elusive whether the multi-system malformations result from failure of mesoderm formation, subsequent patterning processes, or cell fate decisions during organogenesis. Using the zebrafish model, we then analyzed the expression of a select panel of genes that mark early events in mesodermal development during the segmentation period, including *ntl* (axial mesoderm), *myoD1* (paraxial mesoderm), *cdh17* (pronephric intermediate mesoderm), *pax2a* (pronephric intermediate mesoderm), *hand2* (anterior lateral plate mesoderm), and *nkx2.5* (anterior lateral plate mesoderm). WISH in WT and mutant zebrafish embryos indicated that the early mesoderm was successfully subdivided into medio-lateral domains following *med14* deletion (Fig. S3A–S3F). Of note, heart jogging, indicated by the expression pattern of *cmlc2,* was indistinguishable between the WT and *med14* mutants at 30 hpf, while a defect in heart looping in the mutant embryos was evident at 48 hpf (Fig. 1A; Fig. S3G). This finding implies that Med14 is required for cardiac development at later stages.

Studies on fate mapping and knockout phenotypes in mouse limbs have highlighted the crucial role of *Hox13* paralogues in forelimb formation. Mice lacking *Hoxa13* and *Hoxd13* in their limbs failed to develop wrists and digits.^33^^,^^34^ WISH in *med14 Δ17* zebrafish embryos at 48 and 60 hpf demonstrated reduced expression of *hoxa13a* and *hoxd13a* (Fig. 2A–D). Live fluorescence imaging in transgenic zebrafish showed that chondrogenesis in the pectoral fins was strongly compromised, as indicated by a dramatic reduction in the number of *col2a1a*-positive chondrocytes in the pectoral fin in *med14* mutant embryos (Fig. 2C).

We next set out to examine notochord development, as this is instrumental for the proper formation of the vertebrae. The expression of *col8a1a* and *col11a2*, both of which encode integral components of the notochord sheath, was reduced in *med14* mutants at 26 hpf (Fig. 2D). Hematoxylin/eosin staining indicated that the *med14* mutant notochord comprised vacuolated cells of irregular shapes at 48 hpf (Fig. 2E). To closely observe the phenotype of notochord vacuoles in *med14* mutants, we labeled the vacuoles with BODIPY TR methylester dye (MED). Confocal imaging demonstrated that the vacuoles in *med14* mutants were fragmented and generally smaller than those in the WT at 48 hpf (Fig. 2F). In addition, the expression of *vcanb*^35^ and *nkx3.*2^36^, which are involved in the development of the sclerotome that later gives rise to the vertebrae,^37^ decreased upon the loss of *med14* (Fig. 2G and H).

Blockage of the cloaca was evident in the *med14* mutants. To test whether the loss of Med14 affects key events in cloaca formation, we analyzed the expression of early cloaca markers in the *med14* zebrafish mutant embryos. Evidently, the expression of early cloaca markers, *evx1* and *prdm1*, was reduced in the mutants at 24 and 48 hpf (Fig. 2I and J).

We demonstrated that renal function is impaired in the *med14* mutants; to further characterize developmental defects at the molecular level, we examined the expression of known markers of nephrogenesis in *med14* mutant embryos using WISH. *wt1a*^38^ was considered a marker for podocyte progenitors, while *slc4a4*,^39^ *trpm7*,^40^ and *slc12a3*^41^ were used to label the proximal straight tubule (PCT), distal early tube (DE), and distal late tube (DL), respectively. Compared to WT embryos, *med14* mutants exhibited a marked reduction in these markers at 24 and 48 hpf (Fig. 2K).

Taken together, the above results indicate that *med14* is dispensable for early mesoderm formation and subsequent medio-lateral patterning, but crucial for cell fate decisions during organogenesis.

### A novel *MED14* variant identified in a patient diagnosed with VACTERL association

Given the multi-organ malformations observed in the *med14 Δ17* zebrafish mutants, we contemplated the possibility that MED14 might be involved in the etiology of VACTERL association. We screened patients with multiple congenital anomalies and discovered a 2-month-old boy with a variant of the *MED14* gene who had undergone colostomy due to congenital imperforate anus confirmed after birth (Fig. 3). Thoracic computed tomography (CT) indicated that he had mesocardia (Fig. 3A). Physical examination detected congenital polydactyly of the right hand; and X-ray examination further showed a redundant finger located on the lateral side of the right thumb (Fig. 3B). About 60%–95% of patients with VACTERL association present with congenital vertebral malformations^42^; indeed, lumbosacral vertebrae CT of the patient indicated partial loss of the sacrum (S3–S5), and complete absence of the coccyx (Fig. 3C, right). In line with past observations showing a spectrum of renal phenotypes in patients with VACTERL association,^13^^,^^43^ the patient presented with congenital imperforate anus (Fig. 3D, left) and hypospadias (Fig. 3D, right). Colon contrast examination after the injection of contrast medium from previous colostomy and simultaneous cystourethrography indicated that the patient suffered from both rectourethral fistula, an abnormal connection between the rectum and the urethra (Fig. 3E, left), and vesicoureteral reflux (Fig. 3E, middle). During the anoplasty, the colonoscopy inserted from the previous colostomy further indicated the orifice of the rectourethral fistula (Fig. 3E, right). Based on these findings, the patient was diagnosed with VACTERL association.^25^ Whole-exome sequencing for the patient revealed a missense variant of *MED14* located on the X chromosome, where an adenosine was replaced with a guanine, resulting in an isoleucine instead of a valine at the 550th amino acid of the MED14 protein (c.1648A>G; p.I550V) (Fig. 3F). We further examined the inheritance pattern of this variant using Sanger sequencing of peripheral blood collected from the proband and his family, and found that the proband’s mother had one allele of this variant. His maternal grandfather also carried the same *MED14* variant but was asymptomatic (Fig. 3F and G), which is consistent with previous reports indicating great variability in the expressivity of disease-associated genes in VACTERL association.^42^

The I550V variant identified in the proband is among a stretch of amino acids that are highly conserved from zebrafish to human (Fig. S4A), suggesting that it might bear functional significance. To directly assess the role of this VACTERL-associated variant of MED14 in disease pathogenesis and embryonic development, we introduced the same Ile-to-Val variant to the conserved isoleucine residue in the mouse *Med14* gene using CRISPR/Cas9 genome editing on a C57BL/6J background to create the MED14-I556V mutant strain (Fig. S5). Examination of the visceral organs showed mesocardia in approximately 20% of all the mutant mice (Fig. 3H; Table S1). The digit length was also reduced in the MED14-I556V mutants (Fig. 3I and J). Micro-CT scanning revealed the absence of the coccygeal vertebrae in the mutant mice, similar to the observations in the proband (Fig. 3K). Hematoxylin/eosin and periodic acid-schiff (PAS) staining indicated a marked reduction in glomerulus size in about 20% of the MED14-I556V mice (Fig. 3L and M; Table S1). These findings imply a crucial role of the MED14 variant in the manifestation of VACTERL-like symptoms *in vivo*.

### The I550V variant impairs the developmental function of MED14

**Figure 4:**
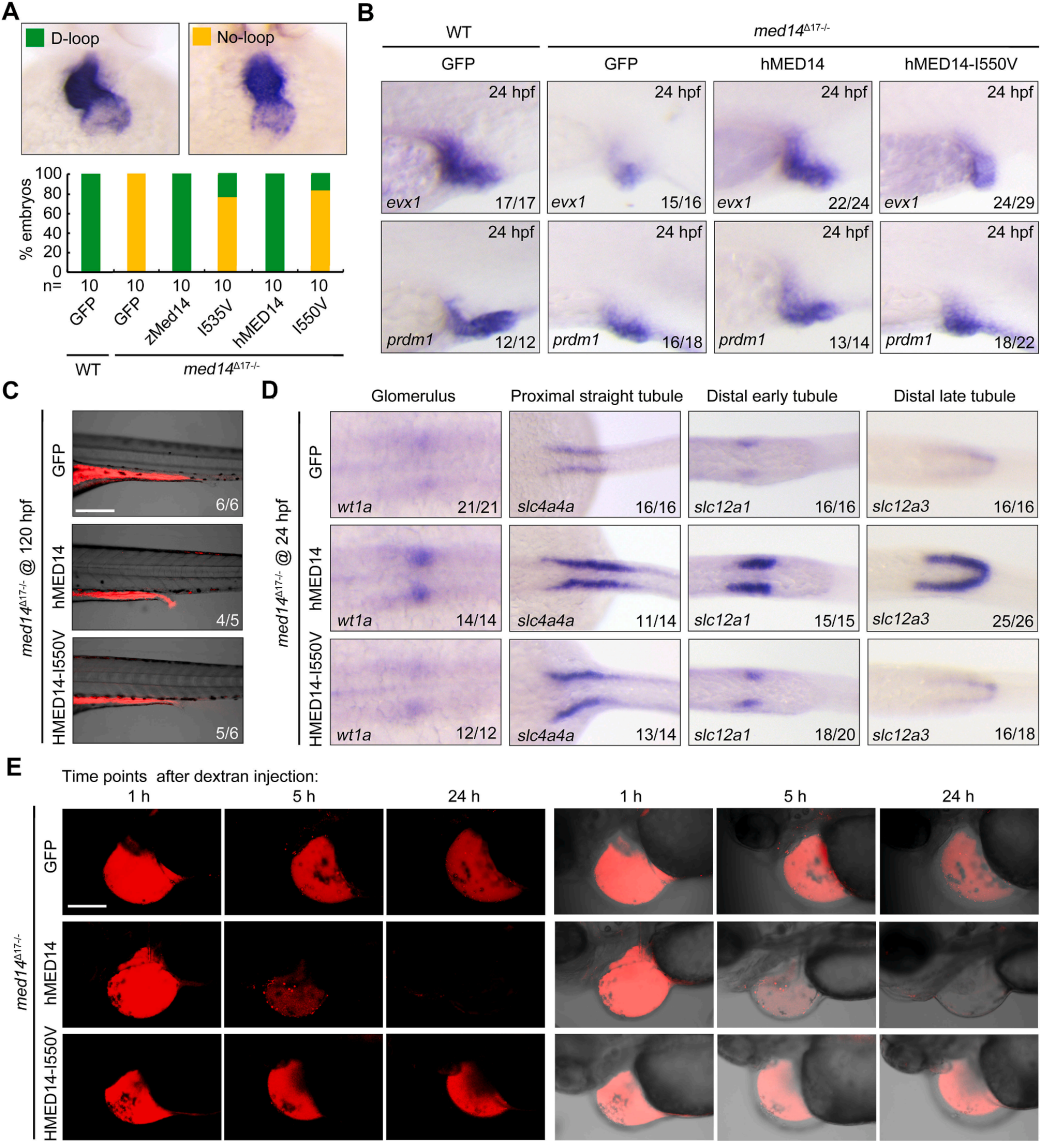
The expression of marker genes for organogenesis from the mesoderm was rescued by WT but not I550V MED14. **(A)** Representative images of heart looping defects in *med14* mutants (upper panel) and quantification of rescue experiments showing the percentage of embryos with the corresponding heart looping phenotypes (lower panel). The WT or *med14* mutants were injected with mRNAs of the indicated genes. Heart looping was assessed with WISH probing for *cmlc2* at 48 hpf. I535V–zMed14-I535V; I550V–hMED14-I550V. Green–D-loop; yellow–No-loop. **(B)** Defects in cloaca formation in *med14* mutant embryos were rescued by the injection of mRNAs of WT hMED14 but not hMED14-I550V. ISH probing for markers of early cloaca formation, *evx1* and *prdm1*, in WT embryos or *med14* mutants injected with the indicated mRNAs. **(C)** Cloaca function was restored in *med14* mutant embryos by the injection of mRNA encoding WT hMED14 but not hMED14-I550V. Excretion of rhodamine-dextran via the cloaca was examined for *med14* mutants upon the injection of the indicated mRNAs. **(D)** Early kidney development was restored in *med14* mutant embryos by the injection of mRNA encoding WT hMED14 but not hMED14-I550V. WISH probing for markers of early kidney development, *wt1a, slc4a4a, slc12a1* and *slc12a3*, in WT embryos or *med14* mutants injected with the indicated mRNAs. **(E)** Expression of WT but not I550V-hMED14 restored kidney function in *med14* mutants. The *med14* mutants expressing the control (GFP), WT (hMED14), or mutant (hMED14-I550V) were injected with 40-kDa rhodamine-labeled dextran via the cardiac venous sinus. The fluorescence intensity of each fish was measured at baseline and after 1, 5, and 24 h.

To further corroborate the functional impact of the I550V variant, we injected WT or mutant (I550V) human *MED14* (*hMED14*) mRNA, as well as WT (*zMed14*) or the corresponding mutant zebrafish *med14* (*zMed14*-I535V) mRNA, into *med14 Δ17* mutant embryos; western blotting confirmed the expression of the injected mRNAs, showing that their expression levels in *med14 Δ17* mutant embryos were fairly consistent with those of endogenous Med14 in WT embryos (Fig. S4B–S4D). Upon mRNA injection, the heart looping defects in *med14 Δ17* mutants were effectively rescued by the expression of WT hMED14 or zMed14 but not by hMED14-I550V or zMed14-I535V (Fig. 4A). In addition, the expression of early markers and the function of the cloaca in *med14* mutants were restored with WT *hMED1*4 mRNA injection but not with mutant *hMED14-I550V* mRNA (Fig. 4B and C; Movies S3–S5). Similarly, the ectopic expression of WT hMED14 but not the I550V mutant ameliorated the accumulation of fluorescent dextran in the pericardial region, suggesting that defects in kidney development were rescued by restoring Med14 function (Fig. 4D and E). Taken together, the expression of WT Med14 rescues VACTERL-like symptoms in *med14* null mutants while the expression of the point mutant Med14 fails to do so, suggesting that the point mutation disrupts Med14 function during development, which ultimately leads to the manifestation of the plethora of congenital anomalies observed in VACTERL association.

### *MED14* point mutation disrupts the integrity of the Mediator complex

**Figure 5:**
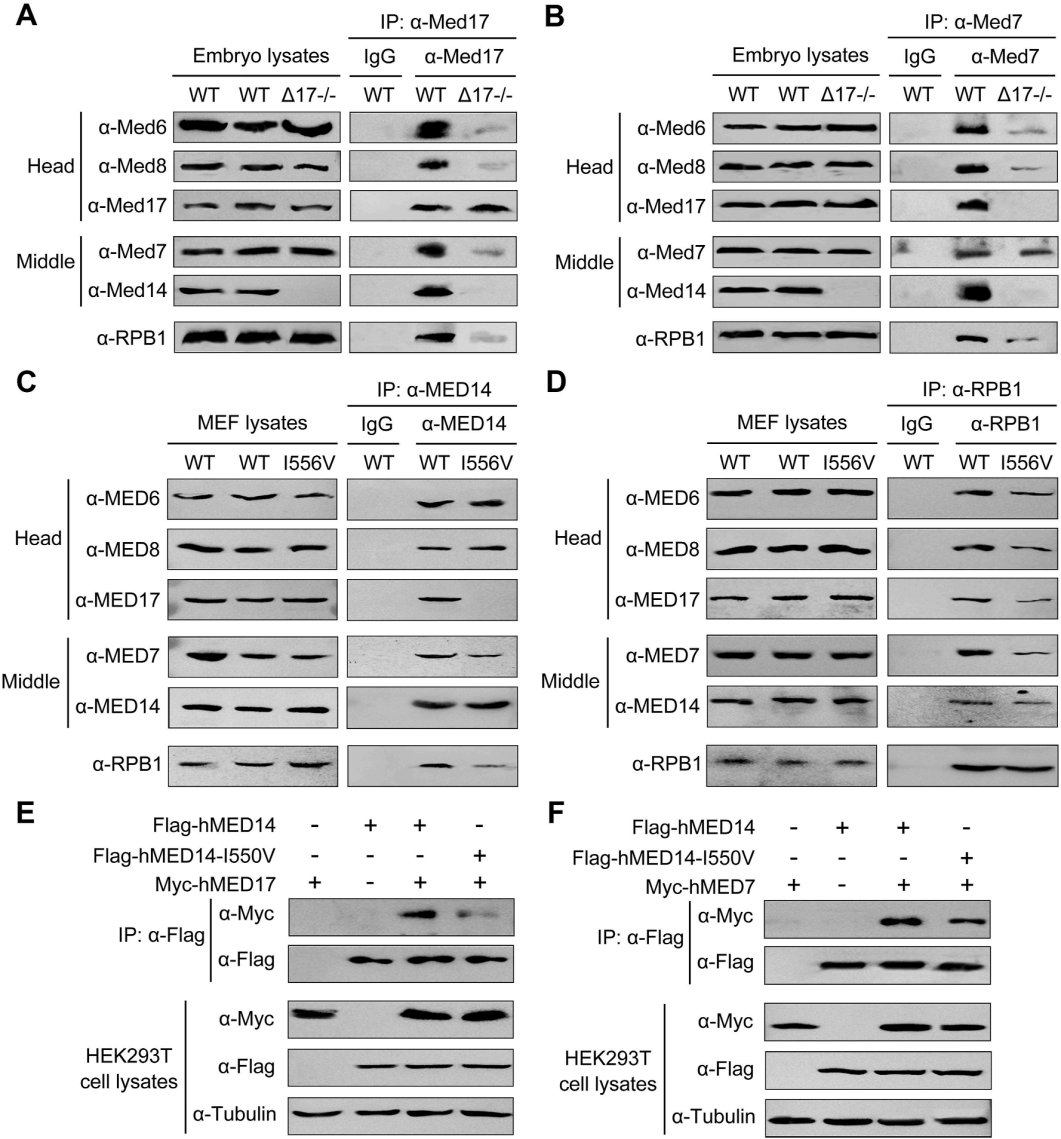
The interactions between MED14 and MED7 or MED17 are disrupted by the Ile-to-Val mutation. **(A, B)** Co-immunoprecipitation revealed disrupted assembly of the Mediator complex in zebrafish *med14* KO mutants. *Med14* mutant embryos were lysed and Co-IPs were performed with (A) anti-Med17 and (B) anti-Med7 antibodies, respectively. **(C, D)** Co-immunoprecipitation revealed compromised interactions of (C) Med7/17 or (D) RNA Pol II with the Med14 point mutant. MEF cells were isolated from WT and I556V mice at E12.5. **(E, F)** IP revealed that the I550V mutation disrupted the interactions between hMED14 and (E) hMED17 or (F) hMED7. Flag-tagged WT or I550V mutant hMED14 was co-expressed with Myc-tagged hMED17 or hMED7 in HEK293T cells. IP pull-down was performed with an anti-Flag antibody, and the pull-down of Myc-tagged hMED7/17 was assessed.

To investigate the molecular mechanism underlying the pathogenesis of VACTERL driven by this MED14 variant, we first assessed the impact of *med14* deletion on the integrity of the Mediator complex using immunoprecipitation (IP). The Mediator complex consists of four submodules including the Head, Middle, Tail, and kinase modules; the former two modules are critical for the interaction of the Mediator with RNA Pol II.^3^ Among the subunits in the Head and Middle modules, MED6, 7, 8, and 17 have been shown to directly interact with MED14.^7^ We therefore performed IP experiments using antibodies targeting either Med17 from the Head submodule or Med7 from the Middle submodule in *med14* mutant zebrafish embryos. Upon the loss of Med14, the interaction between Med17 or Med7 and other subunits of the Mediator, including Med6 and Med8, was dramatically reduced (Fig. 5A and B). Similarly, probing for the largest subunit of RNA Pol II, Rpb1, in Med17/Med7 IP indicated that the interaction between the Mediator and RNA Pol II was also severely compromised in *med14 Δ17* embryos (Fig. 5A and B). Our findings indicated a significant reduction or near absence of Mediator core subunits in the KO mutants, along with a weakened interaction between Mediator and RNA Pol II upon Med14 deletion.

To examine the impact of the MED14 point mutation on the Mediator complex, we first compared the protein stability of WT and mutant forms of zMed14, mMED14, and hMED14 (I535V, I556V, and I550V, respectively) by ectopically expressing these proteins in HEK293T cells. No significant differences in protein levels were observed, suggesting that the point mutation did not affect MED14 protein stability (Fig. S6A and S6B). To further test this in an endogenous setting, we assessed the protein levels of endogenous Med14 in primary mouse embryonic fibroblasts (MEFs) isolated from WT or MED14-I556V mice at E12.5 (Fig. S6C and S6D). In accordance with what we observed in the over-expression experiment, protein levels were consistent for WT and I556V-MED14, confirming that the point mutation does not affect Med14 stability. Next, we tested whether the mutation disrupts the protein–protein interaction between MED14 and other subunits in the Mediator complex. Co-IP experiments showed no difference in the interaction between WT/mutant MED14 and MED6/MED8 (Fig. 5C). However, the interaction between MED14-I556V and MED7 or MED17 was greatly reduced (Fig. 5C). Importantly, the MED14-I556V variant also had a reduced interaction with RPB1 in RNA Pol II, which was further corroborated in the RPB1 IP, showing that the interactions between RNA Pol II and constituents of the Mediator complex were compromised to various degrees in MED14-I556V MEFs (Fig. 5C and D).

Our results suggest that mutant MED14 has weaker interactions with MED7 and MED17. To test whether this is consistent with that of the human MED14 variant, we ectopically expressed hMED14-I550V in human embryonic kidney 293T (HEK293T) cells and performed Co-IP experiments with either MED17 or MED7 (Fig. 5E and F, respectively). Similar to what was observed for the zebrafish and mouse homologs, the hMED14-I550V variant exhibited a reduced association with hMED17 and hMED7, the latter of which was relatively weak (Fig. 5E and F), while the interactions between hMED14 and MED6/MED8 were unaffected (Fig. S7). These results indicate that the MED14 point mutation affects the integrity of the Mediator by weakening the interaction of MED14 with MED7 and MED17, thereby disrupting its interaction with RNA Pol II. Given the biophysical similarity between the original isoleucine residue and the substitutive valine residue, it came as a little surprise that the variant resulted in a partial breakdown of the Mediator complex. However, similar cases have been reported, in which a single missense mutation without dramatic changes in biophysical property was able to disrupt protein functions.^44^, ^45^, ^46^

### MED14 downstream targets are potential regulators of organogenesis

**Figure 6:**
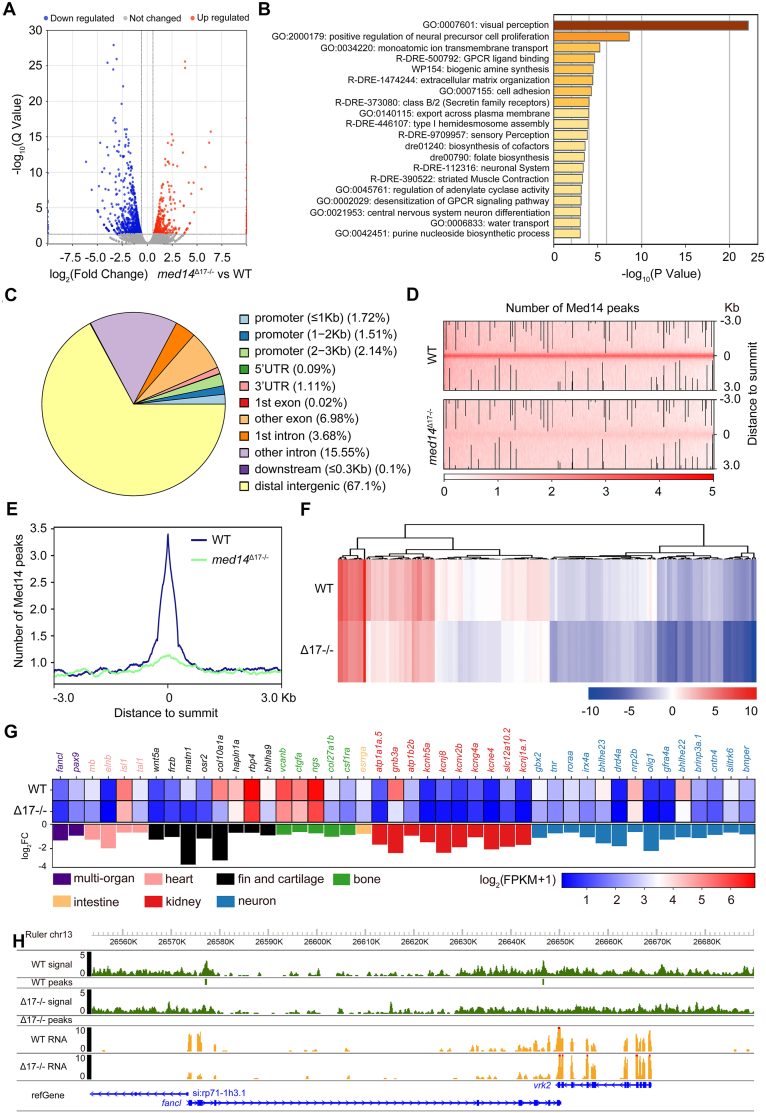
Med14 target genes are involved in the development of multiple organs. **(A)** Volcano plot showing the differentially expressed genes (Fold change ≥1.5, Padj < 0.05) in WT *vs. med14* mutant embryos at 48 hpf. **(B)** GO term enrichment analysis of genes that were down-regulated in *med14* mutant embryos. **(C)** Pie chart showing the genomic distribution of Med14 binding peaks in WT. **(D)** Heatmap showing Med14 ChIP-seq signals in the WT *vs. med14* mutant in the 6 kb region centered on the WT peak summits. **(E)** WT (blue) and mutant (green) aggregated Med14 ChIP-seq signals around peaks proximal to Med14 target genes. **(F)** Heatmap showing the changes in the expression of Med14 target genes as detected by RNA-seq. **(G)** Heatmap (upper panel) showing the expression (log2 (FPKM+1)) of Med14 target genes associated with organ development in WT and Med14 mutant embryos. The lower panel demonstrates the fold change in gene expression (Med14 mutant *vs.* WT). **(H)** Med14 binding signaling (green peaks) and transcript reads (orange peaks) around the *fancl* locus.

To assess the role of MED14 in gene transcription during embryonic development, deep RNA sequencing (RNA-seq) was conducted on WT and *med14* mutant zebrafish embryos at 48 hpf. This bulk RNA-seq revealed 818 down-regulated and 391 up-regulated genes upon the loss of Med14 (Fig. 6A). The number of down-regulated genes is more than twice that of up-regulated genes, indicating the importance of Med14 in Pol II-dependent transcription. Gene Ontology (GO) analysis showed that the most down-regulated pathways are ‘visual perception’ and ‘neural precursor cell proliferation’, which is consistent with a previous report demonstrating that null mutants of *med14* display defective eye and neural crest development.^11^ A plethora of metabolic pathways were found to be down-regulated, such as ‘biogenic amine synthesis’, ‘biosynthesis of cofactors’, ‘folate biosynthesis’, ‘water transport’, and ‘ion transmembrane transport’, which aligns with the observed defects in cloaca development and the renal system (Fig. 6B).

To profile the direct targets of Med14, we performed Med14 chromatin immunoprecipitation followed by sequencing (ChIP-seq) with WT and *med14* mutant zebrafish embryos at 48 hpf. Analysis of the Med14 ChIP-seq data showed a non-random genome-wide distribution of 17,721 peaks corresponding to Med14 binding regions in WT embryos, with more than 30% of the peaks located in intragenic or promoter regions (Fig. 6C). Consistent with previous findings,^47^ a great number of Med14 binding peaks are found in distal intergenic regions, which may harbor enhancer elements that are crucial for the regulation of gene transcription. Heatmap visualization of ChIP-seq signals centered on peak summits (±3 kb) revealed that Med14 binding was severely impaired in *med14 Δ17* mutants (Fig. 6D). We next filtered Med14-target genes by integrating our RNA-seq and ChIP-seq data; we obtained a total of 164 Med14-target genes that were bound by WT Med14 in the intragenic/promoter region or within ±20 kb of the transcriptional start site and were down-regulated upon the loss of Med14. The compiled profile of Med14 ChIP-seq signals confirmed that Med14 binding was almost abolished in *med14 Δ17* mutants (Fig. 6E). Changes in the expression of the Med14-target genes are shown in Fig. 6F, demonstrating a general reduction in the expression of these target genes. For a subset of genes, the reduction in expression was not as dramatic as expected, perhaps due to compensation/contribution from other transcriptional machineries. GO term enrichment analysis indicated that these Med14 target genes are implicated in the development of various organs, including the fin, bone, kidney, and heart (Fig. 6G).^35^^,^^48^, ^49^, ^50^, ^51^, ^52^, ^53^, ^54^ Genes involved in neural development were also enriched, which is in line with the specific expression of Med14 in the midbrain and hindbrain (Fig. 6G).^55^, ^56^, ^57^, ^58^, ^59^, ^60^, ^61^ We have also identified Med14-target genes related to multi-organ development, such as *Fanconi anemia-related* (*fancl*)^62^ and *paired box 9* (*pax9*).^63^ In particular, *fancl* was previously reported to be a VACTERL-related gene.^62^ We further examined changes in the transcriptional landscape and Med14-binding patterns near the *fancl* gene and confirmed the absence of binding and the specific loss of transcriptional activities at the *fancl* locus in *med14* mutants (Fig. 6H). These findings suggest that downstream targets of Med14 are implicated in the organogenesis of multiple organs, which is perturbed in VACTERL association.

**Figure 7:**
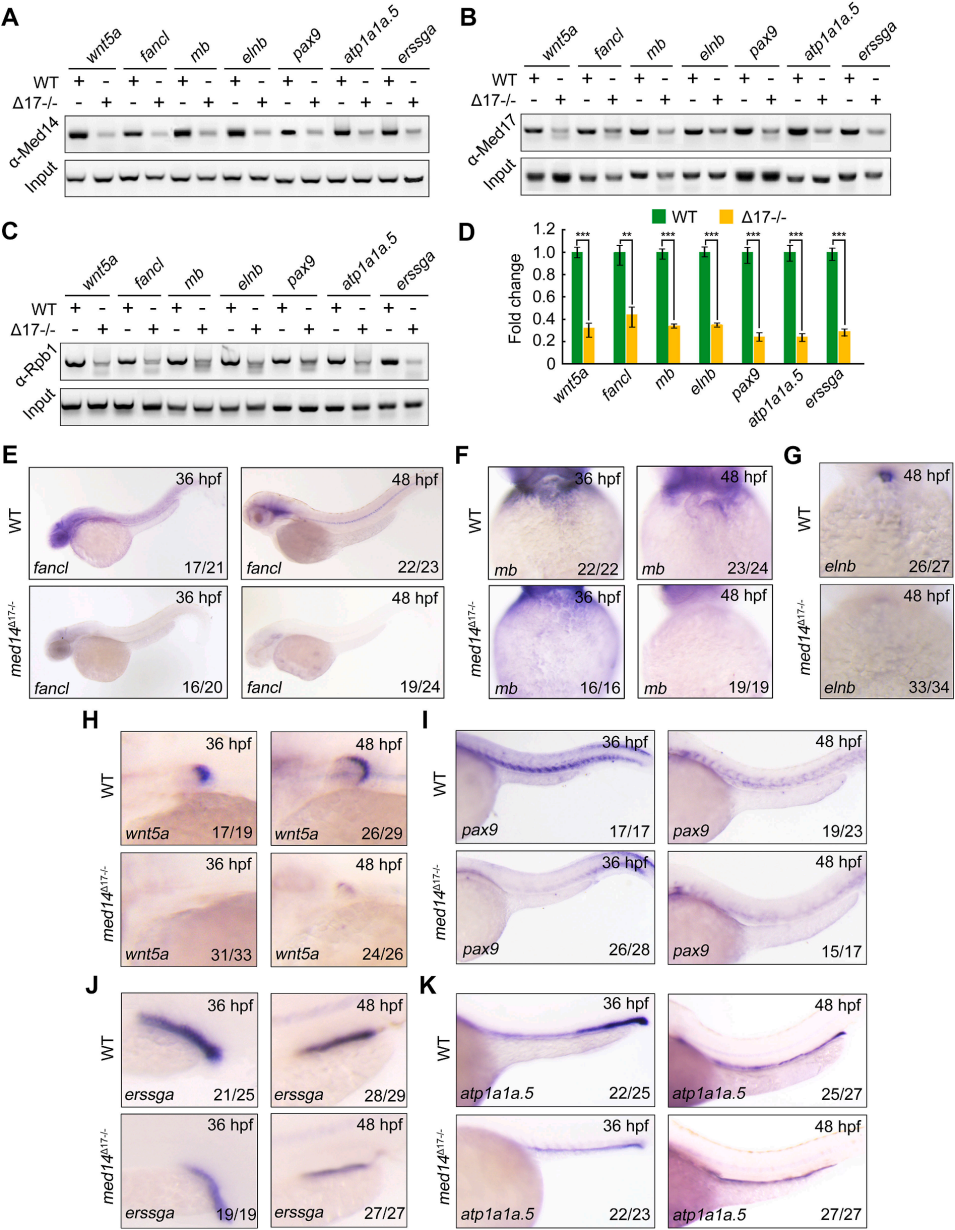
The expression of Med14 targets is reduced in *med14* KO embryos. **(A‒C)** The binding of Med14 (A), Med17 (B), and Rpb1 (C) to the indicated Med14 target genes was tested by ChIP-PCR. **(D)** The expression of target genes was reduced in the *med14* mutants. The expression of the indicated Med14 targets in the WT or *med14* mutants was assessed using qPCR. Statistical significance of the difference in Med14 target expression between the WT and *med14* mutants was determined using a two-tailed unpaired *t*-test. **(E‒I)** WISH was employed to validate these target genes in both the WT and mutants.

To test whether the binding of Mediator and RNA-Pol II to the Med14-target genes is affected upon the loss of Med14, ChIP-PCR using Med17 and Rpb1 antibodies was performed for a select set of target genes chosen based on their specific expression in affected organs and tissues in VACTERL association; and the results indicated a reduced association of Mediator and RNA Pol II with these genes (Fig. 7A–C). Moreover, the expression of these genes was significantly decreased in *med14* mutants, as indicated by quantitative RT-PCR (Fig. 7D). WISH experiments further illustrated the reduced expression of these selected Med14 target genes in the heart, pectoral fin, pronephros, and sclerotome (Fig. 7E–K). Hence, we identified potential downstream genes of MED14 that may participate in the organogenesis of VACTERL-related systems.

Taken together, our discovery reveals that MED14 mutation triggers a spectrum of developmental defects, highlighting its critical role in coordinating gene transcription activities that are required for the proper progression of organogenesis.

## Discussion

MED14 is a central subunit in the Mediator complex, which is a key regulator of eukaryotic transcription.^64^ While studies have linked MED14 to several developmental processes,^10^^,^^65^ a comprehensive characterization of the involvement of MED14 in embryonic development is lacking. Here, we have extensively profiled the series of developmental defects caused by the loss of Med14 function. Importantly, we found that our *med14* loss-of-function zebrafish mutant exhibited phenotypes characteristic of VACTERL association. In addition, we identified a clinically relevant variant of MED14 that might drive the pathogenesis of VACTERL association in both patient and animal models. Our *med14* mutant models displayed normal early mesoderm formation and medio-lateral patterning but showed disrupted cell fate decisions during organogenesis, suggesting that the multi-organ malformations observed in VACTERL association may also be the result of similar cell fate defects. Furthermore, the fact that the *MED14* gene is on the X chromosome provides a potential explanation for the higher prevalence of male patients in VACTERL association.

Mutations in the Mediator complex have been implicated in a variety of human diseases, such as the deletion of *MED15* in patients with DiGeorge syndrome and missense variants of *MED13L* in patients with transposition of the great arteries and intellectual disability.^1^ Interestingly, while all knockout mice of MED subunits are embryonic lethal, mutants of different MED subunits display distinct phenotypes and die at different developmental stages, suggesting that certain MED subunits may harbor functional and temporal specificity during development.^66^ Structural studies have revealed extensive inter-molecular interactions between MED14 and various subunits of the Mediator complex.^2^^,^^67^^,^^68^ In particular, MED14 makes multiple direct contacts with MED17, resulting in an extended MED14-Head module interface.^68^ MED7, a part of the middle module, also interacts directly with MED14.^69^ The I550V variant in MED14 lies within the domain where MED14 interacts with MED17 and MED7, which may explain the specific disruption of the interactions between MED14 and the other two subunits that affects only a subset of downstream functions. Indeed, while both the zebrafish Med14-null mutant model and the mouse Med14-I556V mutant model display many developmental defects that are akin to the clinical manifestations of VACTERL association, the zebrafish mutants exhibited much more severe symptoms than the mouse mutants. Moreover, we found a set of genes involved in neural development among the Med14 targets in zebrafish embryos, while neural defects are rarely observed in human patients diagnosed with VACTERL association.

It has been noted that carriers of the same pathogenic variant in the same family may have vastly different clinical manifestations, ranging from severe physical disability to being completely asymptomatic, as was evident in the family we studied. Several studies have linked the heterogeneous presentation and expressivity of VACTERL association to the complex interplay between genetic and environmental factors.^12^^,^^70^, ^71^, ^72^ An example is the sensitization of otherwise phenotypically normal embryos carrying null variants in the *Haao* and *Kynu* genes with nicotinamide adenine dinucleotide (NAD) depletion.^6^ NAD deficiency during gestation gives rise to mouse embryos with malformations similar to those observed in VACTERL association, while NAD re-supplementation reduces the severity of the symptoms.^6^ Another notable finding is the induction of VACTERL-like symptoms in mouse embryos exposed to adriamycin treatment.^73^ While the Med14-I556V mice show a low penetrance in VACTERL-like symptoms, this animal model will serve as an important tool for dissecting the interplay between genetic and environmental factors in the pathogenesis of VACTERL association.

In summary, our research demonstrated the crucial role of MED14 in organogenesis by maintaining the structural and functional integrity of the Mediator complex. By characterizing the physiological and molecular details of *med14* LOF mutants as well as a clinical variant of MED14, we provide a mechanistic survey of the role of MED14 in embryonic development. These insights are of broad relevance for future research into MED14 functions, VACTERL association, and potentially other human diseases.

## CRediT authorship contribution statement

**Jingwen Liu:** Methodology, Investigation, Visualization, Conceptualization, Writing – original draft. **Xuelai Liu:** Investigation, Methodology, Visualization, Funding acquisition. **Feifei Li:** Methodology, Formal analysis, Visualization, Investigation. **Yujie Wang:** Investigation. **Liping Yang:** Investigation. **Meijia Zhang:** Supervision, Funding acquisition. **Xinyu He:** Project administration. **Ting Zhang:** Project administration, Resources, Supervision. **Ying Yi Zhang:** Writing – original draft, Project administration, Writing – review & editing. **Qiang Wang:** Methodology, Funding acquisition, Writing – review & editing, Supervision, Writing – original draft, Conceptualization.

## Data availability

All the raw sequencing data generated in this study have been submitted to the Genome Sequence Archive in the National Genomics Data Center,^74^ Beijing Institute of Genomics (BIG), Chinese Academy of Sciences (https://ngdc.cncb.ac.cn/) under accession number CRA017693 and can be viewed at https://ngdc.cncb.ac.cn/gsa/s/6r4SAtLb.

## Funding

This work was supported by the 10.13039/501100012166National Key Research and Development Program of China (No. 2020YFA0804000) and the 10.13039/501100001809National Natural Science Foundation of China (No. 32025014, 32330029). This study was also supported by the Beijing Natural Science Foundation (China) (No. 7222015), as well as the 2024–2025 ‘Belt and Road’ International Health Cooperation Project of the Beijing Municipal Health Commission and the World Health Organization Cooperation Center Project (China).

## Conflict of interests

The authors declare that they have no conflict of interests.

## Figures and Tables

**Figure:**
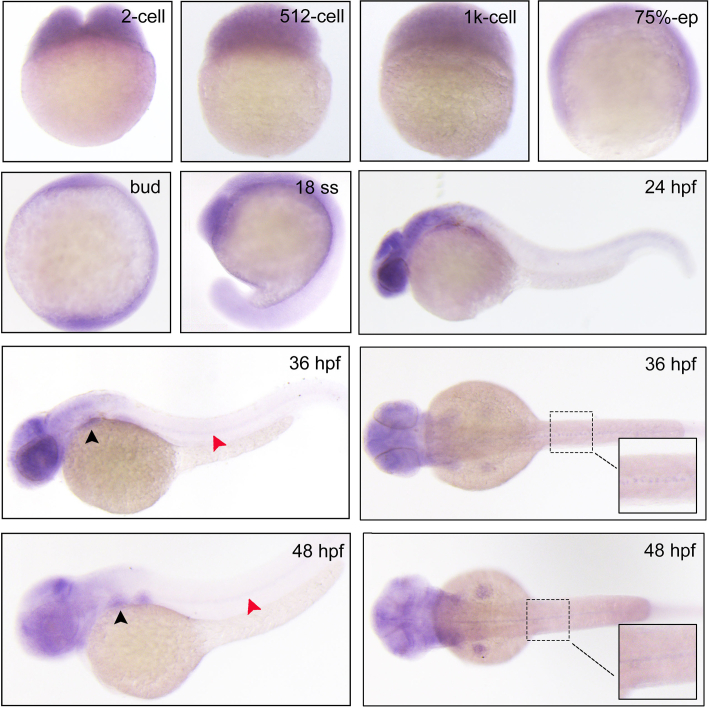
**Figure S1** The expression pattern of *med14* from the two-cell stage to 72 hpf. The expression of *med14* was assessed with WISH at the indicated stages during embryonic development. The black arrow indicates the pectoral fin, and the red arrow indicates the notochord. The expression of *med14* in the notochord at 36 hpf and 48 hpf was highlighted by zooming in on the indicated region of the notochord in the dorsal view of the embryos (bottom right panels).

**Figure:**
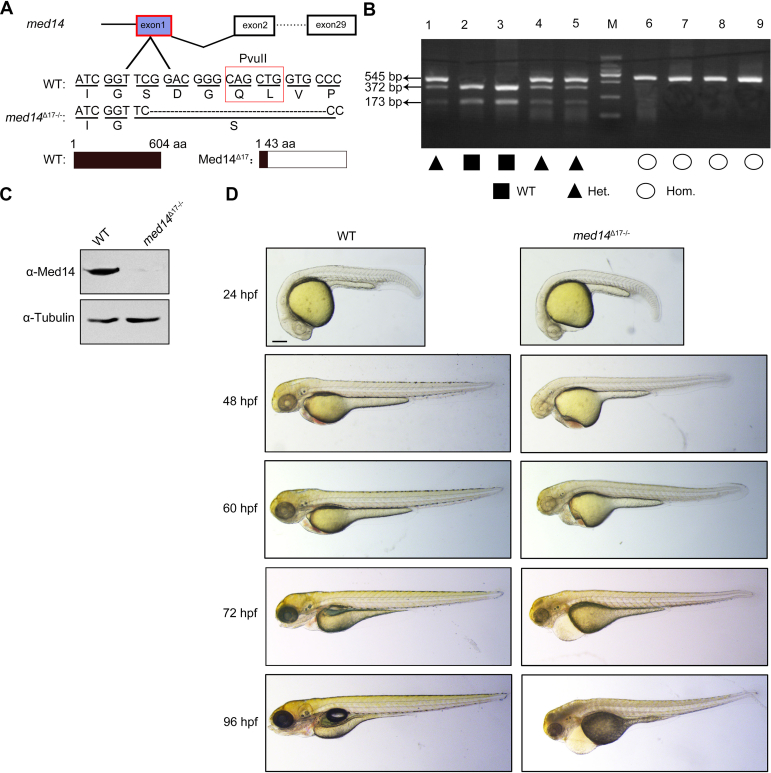
**Figure S2** Construction and verification of zebrafish *med14* mutant. **(A)** Schematic representation of the *med14Δ17* mutation. The red box indicates the PvuII enzyme target site. **(B)** PvuII enzyme digestion of genomic DNA from zebrafish that are WT (square), heterozygous (triangle), and homozygous *med14 Δ17* (circle). **(C)** Levels of Med14 protein in WT and *med14* mutant embryos. WT and mutant embryos were harvested at 48 hpf for western blot using the indicated antibody. **(D)** Live images of WT and *med14Δ17* mutants from 24 to 96 hpf.

**Figure:**
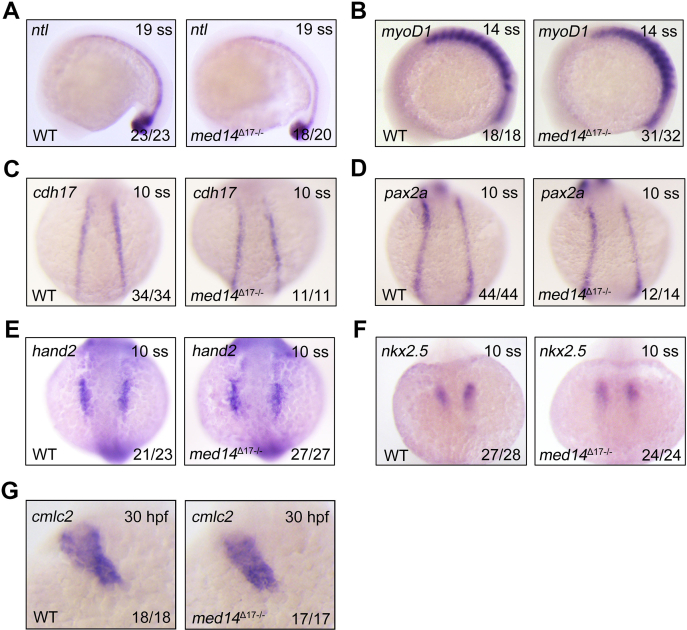
**Figure S3** The specification of early mesodermal lineages is unaffected in *med14* mutants. The expression levels of early mesodermal markers, including **(A)***ntl,***(B)***myoD1,***(C)***cdh17,***(D)***pax2a,***(E)***hand2,* and **(F)***nkx2.5,* were assessed using WISH in WT and *med14* mutant embryos. **(G)** Cardiac jogging, visualized by the expression pattern of *cmlc2*, was assessed in WT and *med14* mutant embryos at 30 hpf.

**Figure:**
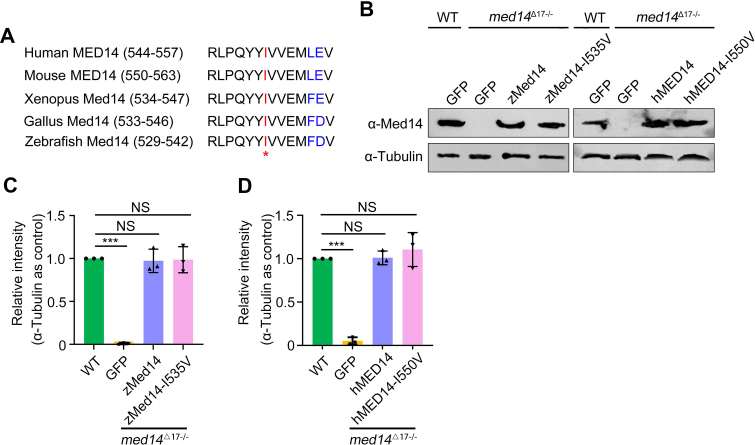
**Figure S4** The affected isoleucine is conserved across species. **(A)** Cross-species alignment shows the conservation of the mutated isoleucine. **(B‒D)** The ectopic expression of WT or mutant zMed14/hMED14 in *med14 Δ17* mutant embryos. The relative levels of Med14 expression in WT or *med14* mutants injected with cDNAs encoding GFP, zMed14 or zMed14-I556V are shown in (C) and are presented as the average of three replicate experiments. The statistical significance of the difference in Med14 expression was measured using a two-tailed unpaired *t*-test. Not significant (NS) = *P* > 0.05 and ∗∗∗*P* < 0.001. The relative levels of Med14 expression in WT or *med14* mutants injected with cDNAs encoding GFP, hMED14 or hMED14-I550V are shown in (D) and are presented as the average of three replicate experiments. The statistical significance of the difference in MED14 expression was measured using a two-tailed unpaired *t*-test. NS = *P* > 0.05 and ∗∗∗*P* < 0.001.

**Figure:**
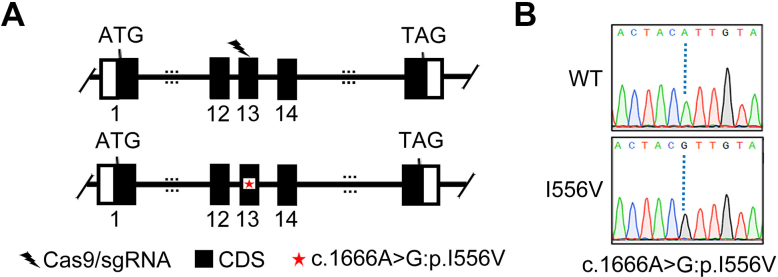
**Figure S5** Construction and verification of the *Med14* mutant mouse strain. **(A)** A mouse strain was created by CRISPR/Cas9, with the target site chosen within exon 13 of the mouse *Med14* gene and a repair template carrying the c.1666A > G:p.I556V point mutation. **(B)** Validation of the point mutation in the *Med14* gene in mutant mice by Sanger sequencing.

**Figure:**
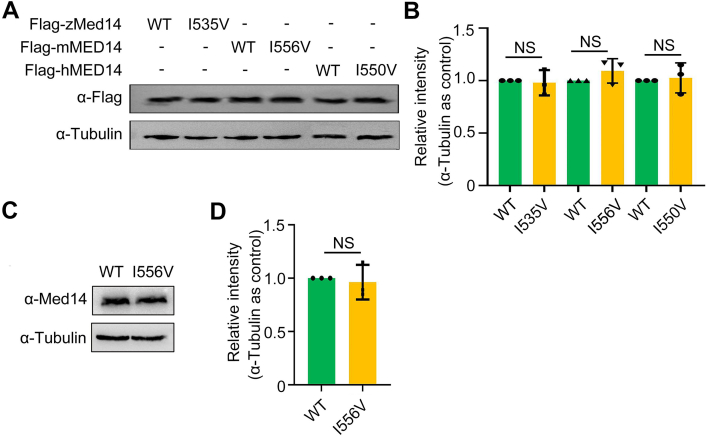
**Figure S6** The Ile-to-Val mutation has no impact on MED14 protein stability. **(A, B)** Immunoblotting was used to assess the expression of Flag-tagged WT/mutant zMed14, mMed14, and hMED14 in HEK293T cells (A). The relative levels of Flag-tagged WT/mutant zMed14, mMed14, or hMED14 expression in 293T cells are presented as thee means of three replicate experiments (B). The statistical significance of the difference in Med14 expression was measured using a two-tailed unpaired *t*-test. NS = *P* > 0.05 and ∗∗∗*P* < 0.001. **(C, D)** The expression of WT/I556V-Med14 in MEFs isolated from WT or mutant mice (C). The relative levels of Med14 expression in WT or I556V-Med14 MEFs are presented as means of three replicate experiments (D). The statistical significance of the difference in Med14 expression was measured using a two-tailed unpaired *t*-test. NS = *P* > 0.05.

**Figure:**
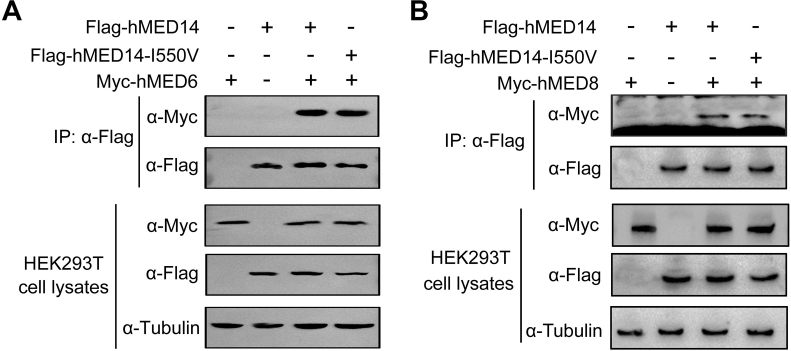
**Figure S7** The interactions between MED14 and MED6 or MED8 are not affected by the Ile-to-Val mutation. **(A, B)** HEK293T cells were transfected with the indicated plasmids and then harvested for immunoprecipitation with an anti-Flag antibody. The interaction between hMED14 and hMED6 (A) or hMED8 (B) was detected by western blot.

## Appendix A Supplementary data

Multimedia component 1 **Table S1** Ratio of mutant phenotypes in I556V-MED14 mice. Multimedia component 1

Multimedia component 2 **Table S2** Primers used for qRT-PCR. Multimedia component 2

Multimedia component 3 **Table S3** Primers used for ChIP-PCR. Multimedia component 3
